# Finite Element Method for Freezing and Thawing Industrial Food Processes

**DOI:** 10.3390/foods10040869

**Published:** 2021-04-16

**Authors:** Tobi Fadiji, Seyed-Hassan Miraei Ashtiani, Daniel I. Onwude, Zhiguo Li, Umezuruike Linus Opara

**Affiliations:** 1 Africa Institute for Postharvest Technology, South African Research Chair in Postharvest Technology, Postharvest Technology Research Laboratory, Faculty of AgriSciences, Stellenbosch University, Stellenbosch 7602, South Africa; 2 Department of Biosystems Engineering, Faculty of Agriculture, Ferdowsi University of Mashhad, Mashhad 91779-48974, Iran; se_mi139@mail.um.ac.ir; 3 Empa, Swiss Federal Laboratories for Materials Science and Technology, Laboratory for Biomimetic Membranes and Textiles, Lerchenfeldstrasse 5, CH-9014 St. Gallen, Switzerland; daniel.onwude@empa.ch; 4 Department of Agricultural and Food Engineering, Faculty of Engineering, University of Uyo, Uyo 52021, Nigeria; 5 College of Mechanical and Electronic Engineering, Northwest A&F University, Yangling 712100, China; lizhiguo0821@163.com

**Keywords:** cell structure, ice crystal, phase change, transport phenomena, heating uniformity, numerical simulation

## Abstract

Freezing is a well-established preservation method used to maintain the freshness of perishable food products during storage, transportation and retail distribution; however, food freezing is a complex process involving simultaneous heat and mass transfer and a progression of physical and chemical changes. This could affect the quality of the frozen product and increase the percentage of drip loss (loss in flavor and sensory properties) during thawing. Numerical modeling can be used to monitor and control quality changes during the freezing and thawing processes. This technique provides accurate predictions and visual information that could greatly improve quality control and be used to develop advanced cold storage and transport technologies. Finite element modeling (FEM) has become a widely applied numerical tool in industrial food applications, particularly in freezing and thawing processes. We review the recent studies on applying FEM in the food industry, emphasizing the freezing and thawing processes. Challenges and problems in these two main parts of the food industry are also discussed. To control ice crystallization and avoid cellular structure damage during freezing, including physicochemical and microbiological changes occurring during thawing, both traditional and novel technologies applied to freezing and thawing need to be optimized. Mere experimental designs cannot elucidate the optimum freezing, frozen storage, and thawing conditions. Moreover, these experimental procedures can be expensive and time-consuming. This review demonstrates that the FEM technique helps solve mass and heat transfer equations for any geometry and boundary conditions. This study offers promising insight into the use of FEM for the accurate prediction of key information pertaining to food processes.

## 1. Introduction

The ability to provide sufficient food quality, quantity, and safety for the growing global population, which is predicted to rise above 9 billion in 2050, is an enormous challenge [1]. Food production and agricultural productivity will have to increase by more than 70% to meet future demands, including dietary energy needs [2,3,4,5]. Addressing energy gaps can reduce demand, lessen the current food waste level, or increase food availability through processing [6]. From primordial times, food preservation has been a link between the food chain and agricultural production with the timely provision of food to people in the required form [4,7]. Although food preservation definitions vary widely, from a general perspective, food preservation refers to any intentional change/action in a food occurring between the origin and point of consumption [7]. Some examples of common industrial processes used in food preservation include freezing, thawing, cooling, milling, heating, drying, extrusion cooking, and fermentation; however, these processes greatly affect the quality of food [8,9,10,11].

The quality index of agricultural and food commodities comprises attributes that facilitate consumer acceptance or rejection [12,13]. During food preservation, some changes occur to the food’s microstructural, nutritional, and sensory properties, which could be detrimental or beneficial to the resulting food quality [14]. Consumer perceptions of foods are influenced by numerous factors, such as texture quality, shape, appearance, and nutritional content, and these factors are strongly impacted by food processing and preservation [1,15,16,17]. Hence, food preservation has become progressively diverse and challenging with the continuous alteration of food preservation techniques, as well as the adoption of new technologies such as freezing, thawing, thermal sterilization, irradiation etc. [1,18,19,20]. Besides, utilizing the advantages of these technologies, including understanding and controlling the complex process–structure–function relationships, offers the possibility for science-based development of tailor-made foods [21]; however, understanding these processes using physical principles could pose a real challenge due to the variability of food materials that are subjected to an array of processing steps [22]. This complexity is considerably increased for freezing and thawing processes in which the phenomenon of phase change occurs as compared to other food processes.

Two broad approaches, experimental and analytical, are often used to better understand and describe various food processing methods. Experimental approaches emphasize the simulation of food processing operations in laboratory- or pilot-scale scenarios. This could be time-consuming, difficult, and expensive, and the correlations used to describe the processes are usually empirical or semi-empirical [23]. Although there may be a paucity of generalized theoretical descriptions of the processes in experimental approaches, these descriptions are indispensable in terms of validating an analytical approach, particularly in cases where methodical laboratory experiments can be established [24,25].

Analytical approaches can be categorized into exact solutions, approximate closed-form solutions and numerical solutions. Exact solutions represent a significant simplification of the complex practical problems and provide qualitative information about trends and orders of magnitude for variables and the associated rates of change, such as heat flux [22]. Approximate closed-form solutions attempt to solve real-world problems, although they limited by some factors such as regular geometrical shapes of food materials, essential or natural types of boundary conditions, and steady or unsteady solutions in one or two dimensions only [22].

The advent of numerical modeling in recent times, with solutions representing a rich tapestry of mathematical physics, numerical methods, user interfaces and futuristic visualization techniques, has become a powerful and prevalent tool in many industries, including the food industry [24,25,26]. The increase in the development of computational resources has supported the development of numerical modeling over the years, where numerical modeling methods have been used as alternatives to obtain approximate solutions to problems when an analytical solution cannot be developed [27]. Some of the benefits of numerical modeling in the food industry include analyzing food processes to better understand the complex physical mechanisms involved, assessing food processes to ensure product quality and designing and optimizing food process systems, as well as controlling food processes by utilizing different suitable predictive models [28].

Finite element methods (FEMs), finite difference methods (FDMs) and finite volume methods (FVMs) are the most extensively used numerical methods for solving fluid dynamics and heat and mass transfer problems. Detailed information about these numerical methods can be found in the work of Peiró and Sherwin [29]. These methods are difficult to compare, and this is largely due to the many variations. FVMs and FDMs are based on discrete solutions, while FEMs provide continuous solutions. To summarize, a FDM could easily form a set of discrete equations to solve partial differential equations of mass and heat transfer. The FEM is more effective for heterogeneous products with irregular geometry and complex boundary conditions as compared with a FDM. It is hard to vary the properties from node to node when using FDMs where changes in physical properties occur [30], and irregular nodal spacing imposes drastic constraints on the allowable time steps [31]. With a FVM, the computational region is divided into a series of non-repetitive control volumes around each network’s grid point. Integrating differential equations for each control volume results in a set of discrete equations [32].

Food preservation and processing methods involving structural and physical properties and transport phenomena introduce an additional degree of complexity with processing constraints related to the functional, nutritional and organoleptic properties [33]. Over the years, the development of food processing technologies and strategies has been modeled to understand the different complex mechanisms involved in food processing. The need for convenient and high-quality food products for consumers has continuously increased. An early review was carried out by Puri and Anantheswaran [22] for the application of a FEM in food processing; however, since FEMs have been proven to benefit the food processing industry, fast development has taken place in the past few years in this regard. In this study, we discuss the state of the art regarding the research, application, and potential of FEMs in food preservation and processing with an emphasis on the freezing and thawing processes from the past 10 years.

## 2. Background for Numerical Modeling in Food Preservation and Processing

Numerical modeling for food preservation and processing is either physics-based or observation-based. Models based on observation begin with experimental data to build the model and are empirical in nature. The physics-based models depend on universal physical laws to describe the physical phenomena of interest and are preferred for predictive models in many areas, including food preservation and processing [33,34,35]. Numerical or mathematical modeling is extensively used in many areas to analyze or improve an existing process and design and develop new concepts. Numerical modeling in food processing principally relies on the fundamental physical mechanisms governing a process that helps provide a basic definition of the process [36,37]; however, these models may need to be validated with experimental data, although they do not have to be available prior to the models. Once a model is validated, it can evaluate the effects of the operating and design parameters involved in the food process [23].

Numerical models have improved understanding of physical phenomena such as heat and mass transfer, fluid flow, and mechanical deformation during food preservation and processing and have been used to redesign or optimize existing food processes [38,39,40]. For instance, predicting temperature distribution in food products would require a heat transfer equation solution [41]. Besides, solving mass transfer equations is crucial in modeling the movement of moisture in food products [42]. Additionally, in a process where the textural properties, sensory attributes, and destruction of nutrients or pathogenic microorganisms is of interest in modeling, reaction kinetics and structural mechanics would play a crucial role [33]. Complex processes can involve several equations for different variables of interest that could be coupled [38]. This means that a variable in an equation could be dependent on other variables. For example, the dependency of local heat flux on local flow velocity, or color change kinetics depending on temperature [38]. Hence, it is worth mentioning that food processing modeling is highly interdisciplinary, integrating engineering, chemistry, predictive microbiology, and reaction kinetics [33,35].

Techniques such as the FEM have fostered the implementation of user-friendly software with the aid of computer-aided design (CAD) platforms to solve model equations in food processes [23], with a reduced focus on the tedious numerical implementation of the algorithms to solve the mathematical equations [38]. A major problem encountered in modeling food processes is the complexity of the combined changes (physical, chemical or biological) during a food process [33]. Others include the primary requirement of compliance with food safety standards and the oversimplification of the food engineering curricula. Important steps in modeling food processes have already been summarized [33].

The FEM is a modeling technique that is effective for irregular geometries, complex boundary conditions and heterogeneous food products. The FEM involves discretizing a large domain into many small elements, developing element equations, assembling the element equations for the whole domain, and solving the assembled equations [24,27]. This technique uses a complex system of points called nodes which make a grid called a mesh. The mesh is programmed to contain the material properties which define how the migration will occur. FEM uses three basic steps to solve a problem: pre-processing, solution or analysis, and the post-processing stages. These stages have been comprehensively discussed in the review by Fadiji et al. [24]. Although the FEM started out as a mathematical technique, most FE analyses now run on commercial software such as COMSOL, ABAQUS, MSC and ANSYS, etc. These software packages have the capability to define complicated geometry and simulate structural, heat transfer and fluid flow problems [43].

## 3. Effect of Freezing on Food Quality

Considering the perishable nature of animal-origin foods, marine products, and plant-based food, preservation technologies like freezing play a crucial role in preserving the freshness and quality of food products [44]. Frozen food products have been extensively appreciated by consumers because of being consistent with a modern lifestyle [45,46], where frozen food products experience an increasing demand of about 20% annually [47]. A freezing process includes three stages: (a) cooling the product from the initial temperature to its initial freezing point (precooling stage), (b) removing the latent heat of crystallization (phase transition stage), and (c) reducing the temperature to the final storage temperature (subcooling stage) [48,49,50]. Nucleation is the initial requirement for freezing, and the occurrence of nucleation requires a driving force called supercooling [51]. During the supercooling period, the temperature rises quickly from the nucleation temperature to the initial freezing point. The ice crystal size is strongly dependent on the degree of supercooling (i.e., the difference between the nucleation and initial freezing temperatures) [44]. Generally, low supercooling degrees cause large ice crystals to form in intercellular spaces and high supercooling degrees result in the formation of smaller ice crystals [52,53]. During freezing, food structures might be under the influence of ice crystal formations within and outside the cells [49]. For food and biological materials, the morphology, size, and distribution of ice crystals presents significant effects on their microstructure. This is due to the irreversible damage to their cellular membrane and tissue which reduces the quality of the frozen products [48]. For example, to produce ice cream with highly desirable sensory characteristics (smooth texture and creamy mouthfeel), it is necessary for the average size of ice crystals to be in the range of 10 to 20 μm. The wide distribution of ice crystals larger than roughly 50 μm creates a coarse or grainy texture in the final product [54]. During freezing, significant changes occur in the microstructure of food as a result of heat and mass transfer, thus affecting product quality stability [55]. During heat transfer, energy is released to the surrounding environment when the product is exposed to a cold temperature. This temperature gradient results in a conductive heat transfer from the center to surface and phase change in the product. In mass transfer, a similar occurrence happens due to the low water vapor concentration in the ambient air compared with the water vapor concentration in the air in equilibrium with the material surface [56].

Both freezing rate and duration have a significant role regarding the growth and distribution of the ice crystals [45]. Slow freezing creates large and irregular extracellular ice crystals, leading to cellular destruction and decreased sensory features [57,58]. The emergence of rapid freezing technologies is highly beneficial to extending the shelf life of foods [59]. Fast freezing processes enhance quality and extend the shelf life of food by preventing microbial growth, reducing water activity, and decreasing chemical and enzymatic reactions [59,60] through the formation of the small and uniform intracellular and extracellular ice crystals in all over the tissue [45,61]. Although high freezing rates protect the tissue structure to a better extent than with slow freezing [62], freezing too quickly leads to mechanical cracking, especially in large products with a high moisture content and low porosity [49,63]. To reduce the freezing duration and control ice nucleation and the formation of countless tiny intracellular ice crystals, new methods such as ultrasound assisted food freezing [64], supercooling technology [63], high pressure freezing [65], magnetic and electromagnetic field-assisted freezing [46,66,67] have been introduced; however, these systems still need to be optimized [44,66,68].

Changes in the quality of food products are not limited to freezing. During storage and transportation, particularly over a long distance, frozen foods experience temperature fluctuations which could lead to moisture and solute migration as well as ice recrystallization, resulting in damage to the texture and taste, undesirable changes in food quality, and surface dehydration [59,69,70,71]. The temperature variations encountered by frozen products are also known as freeze–thaw cycles [50,72]. Improper temperature management in frozen storage results in recrystallization and other physical and chemical changes [73]. Physical changes generally include mechanical damage and cracking, water loss and migration, and freezer burn [62]. At the same time, the chemical changes are mostly manifested as protein denaturation, degradation of pigments and vitamins, lipid oxidation, enzymatic reactions and flavor deterioration [74].

## 4. FEM Applications in Freezing Process

Despite the importance of freezing process in the processing of biological systems, their mathematical descriptions are often difficult to elucidate. This is attributed to the heterogeneous biological nature of foods [75,76,77] since they have cells with different water compositions and morphologies that each have an exclusive diffusion rate [55]. Another reason for this complexity is that the physical and thermal properties of the food products change significantly in phase change region [47,78] to the degree that the error of simple analytical solutions reaches up to 20% [64]. The preference for the food industry is developing methods that could rapidly predict the time/temperature profiles of a product during freezing [45]. To achieve this goal, thermophysical properties including thermal conductivity, density and specific heat must be determined simultaneously during the process [74,79]. Numerical techniques such as the FEM are applicable in modeling heat transfer during freezing process and for predicting the freezing duration. The freezing duration is defined as the time required to reduce the product temperature from an initial value to the desired final temperature [80]. With this method, the plausibility of analyzing the varying thermal properties, food heterogeneity, and the phase change effects over a range of temperature are evident [81]. Hence, modeling the freezing processes of foods becomes crucial when considering the different factors influencing the process. Consequently, allowing for optimization of the freezing process and improving the quality of frozen foods once the models are formulated and implemented correctly and validated [23,59,81]. Table 1 presents the application of a FEM in freezing while highlighting the key findings.

The capability of a FEM in solving complicated shape and severe non-linear problems was demonstrated by Scheerlinck et al. [92] for developing and implementing a FEM for solving nonlinear phase change heat transfer (freezing and thawing) problems with arbitrary three-dimensional (3D) geometries. Huan et al. [84] used a 3D FEM to analyze the freezing and thawing characteristics involved in processing frozen foods to enhance the freezing performance and quality. The authors highlighted food shape and size as well as freezing air velocity and temperature as significant factors affecting the freezing rate. To improve the efficiency of the freezing process, these parameters should be matched with the thermal conductivity of the food. For example, foods with high and low thermal conductivity require high air velocity and low freezing air temperature, respectively.

The freezing process of crab meat packed in plastic pouches and crab claws using a computational FEM was investigated by Dima et al. [83]. Thermophysical properties and experimental heat transfer coefficients measured by differential scanning calorimetry and those determined in the industrial tunnel freezers were used as input parameters in the model. During the freezing process, for the crab meat in pouches, the model allowed for predicting temperature versus time curves while the modeling of the crab claws was done by considering the thermal resistances in series and the irregular bi-dimensional shape of the claws. Additionally, the axial heat flow was considered negligible compared to the transverse energy contribution. There was a phase change in the crab meat during the process while the calcareous layer properties remained constant during the entire temperature change. Equations (1) and (2) were used to describe this process [83]:

(1) $$\rho_{M}\left(T\right)C_{pM}(T)\frac{\partial T}{\partial t}=\nabla .\left(k_{M}\left(T\right)\nabla T\right)\;\mathrm{in}\;\Omega_{M}$$

(2) $$\rho_{C}C_{pC}\frac{\partial T}{\partial t}=\nabla .\left(k_{C}\nabla T\right)\;\mathrm{in}\;\Omega_{C}$$

where ∇ is the gradient, *T* is the temperature, k is the thermal conductivity, $C_{p}$ is the apparent specific heat, and *ρ* is the density. The subscripts “*C*” and “*M*” correspond to the calcareous layer and the crab meat, respectively.

**Figure 1:**
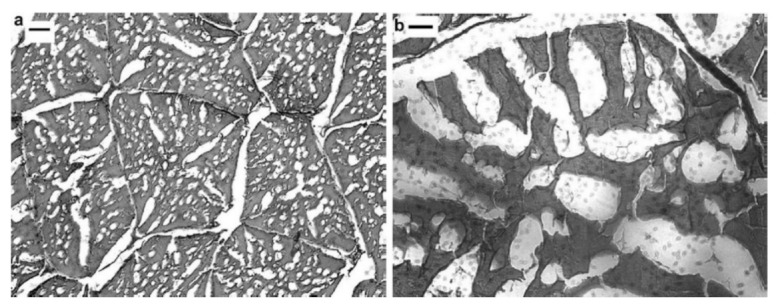
Micrographs representation of crab tissue frozen at different rates and local characteristic freezing times (tc); (**a**) tc = 11 min; bar represents 100 µm; (**b**) tc = 29 min; bar represents 50 µm. Reproduced from Dima et al. [83], with permission from Elsevier, 2014.

In their model validation, Dima et al. [83] established the significant effect of freezing rate on the size of ice crystals in frozen crabs, thereby affecting the microstructural tissue (Figure 1).

Figure 1a,b show small and large intracellular ice crystals with an average ice crystal equivalent diameter of 15 ± 2 µm and 62 ± 4 µm, respectively. In Figure 1a, the micrographs obtained for the frozen crab meat tissue was with an air temperature of −40 °C and air velocity of 4.16 m/s, leading to a freezing time of 11 min. At the same time, for the larger crystals (Figure 1b), there was an observed lower freezing rate with freezing time of 29 min, at an air temperature of −27 °C and air velocity of 3.64 m/s. Here, freezing time was defined as the time elapsed during which the temperature of the sample decreased from the initial freezing temperature (−1.7 °C) to −10 °C. A lower freezing rate (higher freezing time) resulted in larger ice crystals and vice versa, and hence freezing rate directly influences processing time. The quality of frozen food is strongly related to the size of ice crystals. Freeze damage is more likely to occur with larger ice crystals [81], because water expansion during the phase change results in damage to the cell membranes [93,96,97]. The increased volume exerts a greater pressure and mechanical damage on cell membranes, which can crack and cause mass loss during thawing [93].

The study by Sadot et al. [93] used FE simulation based on original enthalpy formation and the latent heat due to the ice crystal radius growth to model an innovative microwave-assisted freezing (MAF) method to improve the quality of frozen products. The model assumed ice crystals to be spherical and have the same radius. Thermophysical and dielectric properties, which evolve between fresh and frozen properties, were modeled according to the ice mass fraction variation. Heat transfer was modeled by using a partial differential equation to solve the heat Equation (3) based on enthalpy formulation in Equation (4), integrating the latent heat of water solidification. Heat generation was calculated using Equation (5) based on the local electric field induced by microwaves.

(3) $$\frac{\partial T}{\partial t}\frac{\partial h}{\partial T}.\rho -\nabla .k\nabla T=Q$$

(4) $$h=\left[Cp_{nf}-\left(Cp_{nf}-Cp_{f}\right)\frac{x_{ice}}{x_{fw}}\right]T+\left[L.x_{fw}-\left(Cp_{nf}-Cp_{f}\right).T_{if}\right]\frac{x_{fw}-x_{ice}}{x_{fw}}$$

(5) $$Q=\frac{1}{2}\omega \varepsilon_{0}\varepsilon^{\prime \prime }{\left|E\right|}^{2}$$

where *h* is the specific enthalpy, *Cp* is the specific heat capacity, *x* is the mass fraction, *T* is the temperature, *L* is the latent solidification heat, *E* is the local electric field, *ω* is the pulsation, *ε*_0_ is the vacuum permittivity, *ε”* is the relative dielectric loss factor, and *Q* is the source term corresponding to the heat generated by microwaves. The subscripts “*nf*”, “*f*”, “*if*”, “*fw*” and “*ice*” correspond to the non-frozen, frozen, initial freezing, freezable water and ice, respectively.

A COMSOL FEM was used to solve the coupled equations numerically in 2D with an unsteady state. The maximum resolution step time was 0.1 s with 1 s of sampling to obtain sufficient accuracy due to the short duration of the microwave pulses. For the mesh sensitivity, the quadrangle mesh size was reduced until a relative RMSD lower than 5% was obtained. The computational time ranged between 30 h and 72 h depending on the configuration.

The model, validated with data from literature, could quantify the temperature oscillations caused by the microwave pulses, and predict a temperature amplitude of about 0.1 °C. Further, the amplitude of the temperature oscillations was found to be related to the pulse duration.

It is worth mentioning that freezing and thawing are heat transfer processes with a phase change. The change in temperature is described as an unsteady state heat transfer process, which is a function of time and location in the frozen process. Temperature distribution can be predicted using the equation for heat conduction (Equation (3)). *Q* is a source term (W/m^3^) referring to a distributed heat source or heat sink depending upon whether the product is being frozen or thawed, respectively. In food products, freezing or thawing does not occur at a constant temperature. The latent heat will be released (during freezing) or absorbed (during thawing) over a range of temperature due to the changes in the concentration of solutes [87].

More recently, Nguyen et al. [82] used a FEM to describe the freezing process of a fish fillet. A coupled heat and mass transfer problem was investigated in a discretized computational domain using the COMSOL solver. The temperature distribution results showed a trend for the decreasing temperature rates to be lowest and highest at the centroid and the four corners of the fish fillet, respectively. The required freezing time predicted by simulation conformed well with experiments. The study formed a basis for future research related to including electromagnetic fields during the freezing process to improve the quality of frozen foods and energy performance, particularly for industrial-scale production. During the modeling of freezing process, solving nonlinear mathematical problems, especially when the thermophysical properties changes with temperature and time, it is without doubt that FE simulation has been proven to be capable of solving the problems faced during food freezing with a high level of precision and reasonable stability [98].

## 5. Effect of Thawing on Food Quality

Thawing is the reverse process of freezing and it is a fundamental step for frozen food products before consumption [99]. Thawing can be defined as the melting process of converting a frozen state to liquid state (i.e., melting), and usually takes place slower than that with freezing [100]. Traditional thawing methods such as water immersion thawing and air thawing are very slow because of the low thermal conductivity of frozen food products and can consequently deteriorate the quality of products [101,102,103,104]. Thawing time, which refers to the time taken to reach a temperature of 1 °C throughout the product [101], is directly related to the risk of pathogenic and microbial contamination in frozen food products [105,106]. Reducing the time to thaw foods will minimize microbial growth, chemical deterioration, excess water loss due to dehydration or dripping, and decrease energy costs [103,107,108,109,110]. During a long thawing process, the quality of the product is significantly deteriorated through protein denaturation, mass loss, and changes in taste, texture, and color [107,111,112]. Therefore, better quality and higher energy efficiency are realized when the thawing time is shorter [113].

To minimize dripping during thawing and deterioration in quality, new technologies have been developed in recent years. These include microwave thawing [102,114,115,116], ohmic thawing [113], high-pressure thawing [61,101], ultrasonic thawing [107,117], radiofrequency thawing [33,108,118], high-voltage electric field thawing [104,111], and far-infrared thawing [112]. Among these methods, microwave thawing presents advantages such as a short thawing time, easy control, fast heat efficiency, and energy saving. These advantages have resulted in the extensive use of microwave thawing, both in industry and at home [112,119,120,121]. Nonetheless, its major problem is non-uniform heating via the uneven distribution of the microwave field inside the food product [114,122].

Ohmic thawing is an innovative method where frozen foods positioned with negative electrons are introduced into a high-voltage electrostatic field and are heated uniformly [123]. The application of ohmic thawing has considerably shorter processing times than conventional methods at the same temperature range [110], yielding thawed foods with higher quality traits, integrity, flavor and nutrient retention [124,125].

In a high-pressure thawing process, frozen foods are thawed at relatively low temperatures using high pressure [123]. The high pressure lowers the melting point and specific heat capacity of ice while increasing the thermal conductivity [101]. By increasing the pressure to 210 MPa, the freezing point of water decreases to −21 °C, and above this pressure level an opposite effect is observed [61,101,123]. Though high pressure-assisted thawing can reduce the thawing time and drip loss, it does have some limitations, where the technique is expensive and causes pressure-induced protein denaturation and meat discoloration [61,126].

With ultrasound techniques, vibration energy generated by ultrasonic waves is converted to thermal energy inside the food product. Although, ultrasound techniques reduce the possibility of water loss, microbial contamination, and protein denaturation, high energy consumption, poor penetration, and localized heating are some of the weak points of this method [61,106,119].

The principles of heat generation by a radio frequency (RF) system are similar to those of microwave system, but they differ in terms of the electromagnetic wave frequencies [108]. During RF thawing, polar molecules begin dipole rotation via field change, and thus electromagnetic wave energy is converted to heat in the food product, leading to thawing [101]. The common challenge for RF heating is non-uniform heating caused by the inevitable uneven distribution of the electromagnetic field between the product and surrounding space (mostly air), particularly for food products with a high moisture content [127,128].

In far-infrared heating, electromagnetic energy collides with and penetrates the product, where it is converted to heat [129]. Although far-infrared thawing can improve the quality of frozen products, the risk of overheating due to an insufficient penetration depth of infrared waves still exists, especially for thicker products [106].

The high-voltage electric field thawing method is a non-thermal method which produces corona wind [61]. Turbulence and vortices produced by the corona wind accelerate the heat transfer in frozen food [130]. This method increases the thawing rate and limits microbial growth and reduces energy consumption [104]; however, the large equipment size and high cost are the drawbacks of this system [101]. Thus, optimization and innovation in thawing systems/processes will help the food industry establish more efficient, safer, cheaper and more convenient methods [13,101,118]. Numerical simulation methods are efficient tools to achieve this goal. For instance, a FEM could help achieve a uniform distribution of an electric field inside a frozen product by increasing understanding of the interactions between electromagnetic radiation and the food matrix in different conditions [127,131].

## 6. FEM Applications in Thawing Process

Table 2 presents some application of FEMs in thawing processes while highlighting the key findings.

With regards to a FEM, Campañone et al. [137] modeled the heating of food in a microwave oven while allowing for the characterization of temperature distribution. Microscopic mass and energy balances were solved considering the electromagnetic interaction with the product. Heat transfer within the product was described by formulating the microscopic energy suggested by Ayappa [138]. The water concentration profile was calculated by formulating the microscopic mass balance proposed by Marra et al. [139]. In the model, the following assumptions were made:

- variable thermal and dielectric properties;
- uniform initial temperature and water concentration within the products;
- no volume change during heating;
- plane plate geometry, 1D heat and mass transfer;
- convective and evaporative boundary conditions for heat transfer.

The validated model was used to study the thermal response of foods with different shapes and operating conditions. The authors proposed incidence wave interference as an effective strategy that produces uniform temperature profiles. At the same time, the processing time was significantly reduced with the use of air convection in combination with microwaves.

One of the challenges encountered in the simulation of RF processes, particularly involving the modeling of thawing, is the variation of thermophysical and dielectric properties in the phase change region, thus inducing further complexity [118]. Besides, the phase change process requirement involves dealing with the evolving large latent heat over a small temperature range [118,122]. Therefore, the modeling of RF thawing process becomes a multi-physics problem regarding dealing with these challenges. Hence, modeling a RF thawing process involves solving coupled heat transfer with electrical field distribution as well as the phase change process [96,108], although most RF processes focus mainly on heating uniformity [140,141,142].

Uyar et al. [118] developed a validated FEM for an RF system to obtain the electrical field distribution inside a RF system and temperature distribution in a frozen product (lean beef meat) while thawing. To determine these distributions, a Fourier heat transfer equation with a generation term coupled with the quasi-static electro-magnetic field equations, in addition to handling the phase change, was solved as shown in Equations (6) and (7). Additionally, the model solved coupled heat conduction and the electric field in a 3D domain with the dependency of thermophysical and dielectric properties on temperature.

(6) $$\rho C_{p}\frac{\partial T}{\partial t}=\nabla .k\nabla T+Q_{abs}$$

(7) $$Q_{abs}=2\pi f\varepsilon_{0}\varepsilon^{\prime \prime }{\left|\bar{E}\right|}^{2}$$

where *ρ*, *C_p_*, *k*, *t*, and *T* are the density, specific heat, thermal conductivity, time, and temperature, respectively, *Q_abs_* is the RF power absorbed per unit of volume by the load, *f* is the frequency of the RF wave generator, *ε*_0_ is the permittivity of free space, *ε*″ is the relative dielectric loss factor of the product and $\left|\bar{E}\right|$ is the modulus of the electric field.

**Figure 2:**
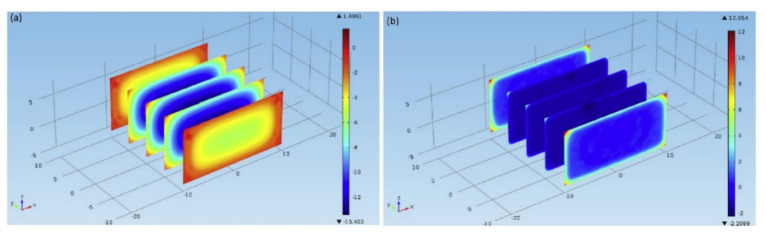
Contour plot showing the temperature distribution of the lean beef at a radio frequency of 50 Ω and thawing times of (**a**) 600 s and (**b**) 3000 s. Reproduced from Uyar et al. [118] with permission from Elsevier, 2015.

The study demonstrated the influence product size and power absorption has on thawing time. Comparisons were made between the conventional natural convection thawing and RF thawing under still air. Increasing the sample size increased the RF power absorption and consequently reduced the thawing time. Figure 2 shows the predicted temperature contour distributions of the frozen product in the *z*-*x* plane at the thawing time of 600 and 3000 s.

This numerical prediction showed that the temperature was rather uniform towards the end of the thawing time, elucidating the importance of the RF wave in obtaining temperature uniformity within the product. A major disadvantage of the RF thawing system is the non-uniform temperature distribution, particularly for high temperatures observed along the surface and corners of the product during thawing. In comparison, the simulation results agree well with the experimental data.

The concept of microwave thawing, although widely available nowadays, is physically complex. A significant advantage is its faster heating and lower energy consumption [143]; however, the high non-uniformity of spatial energy deposition is a causative effect of the electromagnetic properties of a microwave oven. To model a microwave thawing process realistically, solving 3D electromagnetics in a cavity coupled with heat transfer is required, posing computation challenges. Additionally, an increase in varying dielectric properties with temperature results in greater coupled electromagnetic and thermal problems during modeling. To this end, Chen et al. [115] included all the fundamental physics to comprehensively understand the mechanisms of microwave thawing in a cavity by developing a validated model for the microwave heating of a food analog (a tylose cube). The physics properties included (a) the complete electromagnetics, (b) the frozen fraction that continually varies with temperature, (c) the dielectric and thermal properties that continuously change with the frozen fraction and (d) the two-way coupled electromagnetics and heat transfer. The total energy absorption reduced with product thawing, and the heating mode had a negligible effect on the heating uniformity. An increase in the size of a food product results in less uniform heating in the food volume, which ultimately results in changes in corner and edge heating. The validated model proposed a fast and effective way to predict microwave heating rate and uniformity.

The dielectric properties, specific heat, and thermal conductivity as a function of temperature were used as input material properties to simulate the microwave heating of chicken nuggets and steamed bread [135]. The temperature profiles from the model observed at 90 s of heating at 700 W were consistent and corresponded with the experimental results. The authors proposed the applicability of the model in identifying cold and hot regions with a product to ensure quality with respect to temperature uniformity. During handling operations in the cold chain, it is without a doubt that temperature fluctuations and consequent induced heating of the frozen foods could alter the texture, color, flavor, and quality of products. Cevoli et al. [47] used a FEM to investigate the effect of environmental temperature on heat transfer inside packed frozen foods (peas, spinach cubes and grilled aubergines). The capability of the model was adopted in describing the conduction and convection processes inside and on the surface of the packages. The highest temperature values were observed at the bottom of the product in contact with the metallic rack. Hence, the bottom zone was the most critical area for thawing. The temperatures measured at the center and bottom area of the products from the numerical model were in good agreement with the experimental data with a maximum error of approximately 1.8 °C.

Prior to the processing (e.g., slicing, cutting, and dicing, etc.) of meat, the products are thawed or tempered [144]. During thawing, a product’s temperature is increased until the temperature of the coldest region is above the freezing point. In comparison, after tempering, a lower target product temperature should be reached [145].

**Figure 3:**
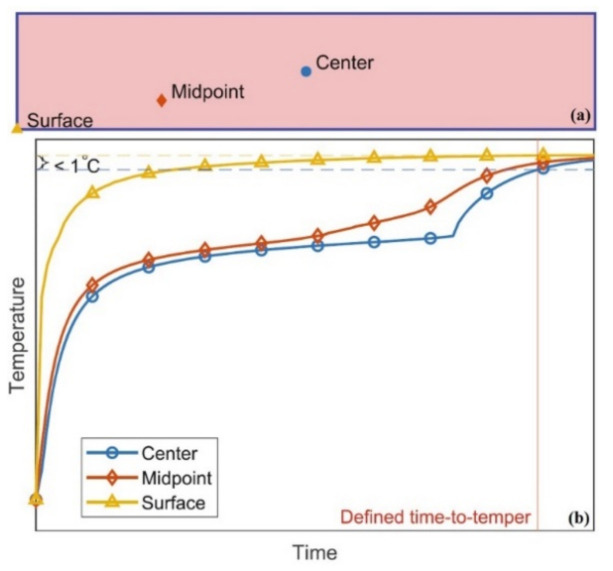
(**a**) Surface, midpoint, and center of the studied sample. (**b**) Tempering time definition based on the temperature distribution. Adapted from Shan and Heldman [136], with permission from the corresponding author, 2021.

Recently, the study by Shan and Heldman [136] focused on evaluating the effects of ambient temperature (*T_a_*), convective heat transfer (*h*), product dimension and product composition on the tempering time of frozen meat (pork). The study used a FEM to simulate the temperature distribution within the frozen product during the tempering process. In the simulation, the product was considered as a homogenous mixture of all components with no drip loss occurring during tempering. From the results, *h* from 200 W m^−2^ K^−^^1^ and above does not significantly reduce the tempering time while more variations were observed on the effect of *h* on tempering time when *h* was between 0 and 200 W m^−2^ K^−1^. In the study, three positions (Figure 3a) were of interest and an illustration of the temperature profile, including the defined time to temper, is shown in Figure 3b.

Shan and Heldman [136] defined the time to temper or otherwise tempering time as the time the frozen product takes to reach a target temperature with a difference in temperature between the surface and the slowest warming location within the product being <1 °C. Figure 3b shows that temperature at the center and surface increases the slowest and fastest, respectively, for a convective heat transfer process. For the product composition: pork loin, pork belly and pork fat, at *T_a_* of 4 °C, the time to temper for the pork fat was smallest while that of the pork loin was greatest. This was attributed to higher water content in the pork loin than the port fat with lower water content. The latent heat needed to be overcome in the pork loin during the phase change is higher with higher water content. Consequently, the specific heat of the product on reaching freezing point is higher, resulting in a lower thermal diffusivity. Additionally, increasing the product thickness increased the tempering time. Besides, the ratio of the thickness increase was lower than that of the time to temper.

## 7. Current Limitations and Future Trends

FEMs play an important role in improving various process operations and have gained momentum in industrial food applications, especially in freezing and thawing processes. Despite the numerous advantages, there is room for further improvement and development. Assumptions made during simulations are often considered to reduce complexity and the computational time of the model, which could adversely affect the accuracy of the model. Some of the assumptions made during the simulation of freezing and thawing of food products include uniform initial temperature distribution throughout the product, material homogeneity [114], uniform distribution of convective heat transfer coefficient around the product [10,134] and ignoring mass and momentum transfer in the product [114,133]. Reasonably limiting assumptions could improve the accuracy of the simulation, although the running calculation could be longer.

Frozen products are handled under various freezing conditions. For instance, the products may be packed in bulk, and modeling freezing process of bulk products, assuming the box full of the products would be a source of error. This is because, in real conditions, there is a head space inside the box. Thus, to improve the accuracy of the model, it is important to include the heat transfer mechanism that occurs in the head space as well as equations to estimate the temperature changes of freezing point, and the thermophysical properties during the freezing process [45].

It is well established that the size of ice crystals affects the quality of a frozen product [81,93]. Larger ice crystals usually result in greater freeze damage [81,146], and this is related to decreased water reabsorption during thawing process caused by smaller specific surface area in large ice crystals [93,147]. Measuring the average size of ice crystals and 2D image data provides little information about the changes in individual crystals and the evolution of their size distribution during frozen storage. Hence, developing 3D models allows for a better description of the evolution of ice crystal size in food products [148].

Another challenge concerning FEMs for freezing and thawing process includes creating irregular geometries of food products in the simulation environment. As such, modeling these processes has been generally developed by assuming a regular shape for the food products. Irregularly shaped products have more curved surfaces, pores, and uneven thickness than regularly shaped products, leading to a non-uniform heating pattern. Additionally, when horticultural products are packed in bulk, the shape of individual products combines, forming a stack with a complex and convoluted shape, affecting the rate of transfer processes [149]. An additional complexity is imposed on creating transport models when a polyliner bag covers the surrounding of the product bulk. Defining the surface topography of the product bulk is difficult in a modeling environment because a polyliner bag is flexible and sticks to edges [149]. Therefore, developing rapid and efficient methods for the 3D reconstruction of irregular food products becomes a necessity. Micro-slicer image processing systems (MSIPSs) and X-ray micro-computed tomography (X-ray micro-CT) are some of the techniques used to measure the 3D structures and distributions of ice crystals in frozen food products [150,151]. The X-ray micro CT method is a non-destructive method that allows complete reconstruction of the object structures without needing sample preparation or chemical fixation [152]. Therefore, this system could potentially be used to visualize and measure the structure of different food materials.

Improving model accuracy is not merely possible by focusing on developing simulation algorithms and developing laboratory methods for model validation [23,24]. Nonetheless, a problem encountered, for instance in freezing and thawing processes is the need to provide explicit data on the thermophysical properties (such as density, thermal conductivity and heat capacity) of a product which due to its anisotropy and lack of homogeneity may be challenging [153]. Most laboratory methods measure thermophysical properties of a product under steady-state conditions; however, these properties change suddenly at a temperature close to the initial freezing point; thus, controlling the error caused by temperature measurements is difficult [154]. Although the freeze time and temperature distribution could be experimentally measured during the freezing process, this is costly, time-consuming, and lacks a complete theoretical description of the process [88], as measuring temperature spot by spot thermocouple is not feasible in a biological structure [47,155]. Furthermore, process design data and new designs in thawing systems are often based on trial and error [102,120]. Due to changes in the properties and structure of components in food products during the thawing process, trial and error methods are costly and time-consuming [115]. They often provide limited information that cannot identify the latent mechanism in the process [121,128].

FEM methods predicts freezing and thawing times more accurately than analytical methods; however, they require the control of numerical error, which depends on parameters like size of grid, change in boundary conditions and material properties, and time step etc. Adequate knowledge of the physical phenomena involved in the processes and familiarization with the physics involved in the modeling will be necessary to set-up a proper model capable of accurately representing the physical model [24,25]. To date, FEMs have been proven to be successful for better understanding the freezing and thawing processes of food products. The continuous advancement and development of more sophisticated software platforms will yield more accurate analyses, improve insights and optimize designs and processes, even though specialized software and equipment are not always cheap.

## 8. Conclusions

The use of FEMs has rapidly developed in different food processing operations, particularly freezing and thawing processes, in order to supply fresh, delicious, safe, nutritious, and minimally processed products into the market. This review has presented a comprehensive summary of recent studies on FEMs regarding freezing and thawing processes for food products. Attention to FEMs has greatly increased, and the scientific literature has shown the promising prospects regarding efficiently predicting the process of heat and mass transfer in food products and freezing and thawing times, including temperature patterns and distributions in frozen foods. The efficacy of applying FEM modeling in simulating irregular geometry, complex boundary conditions and heterogeneous food products has been shown. In combination with FEMs, experimental analyses are crucial to determine model input parameters and validate simulation predictions. It is noteworthy that FE simulations can reduce costs, processing time, and equipment optimization, allowing a more detailed physical visualization of the dynamics that occur during freezing and thawing processes. Continuous advancement and development will help enhance FE simulations to be used as a powerful engineering tool in the food processing industry, especially for freezing and thawing processes.

## Figures and Tables

**Table 1:** Examples of finite element method application in food freezing. FEM: Finite element model.

Reference	Material	FEM Analysis Tool	Dimension	Resolution (type/size)
[82]	Fish fillet slice	COMSOL	2D	NA
	Study purpose: To predict the freezing time and model the freezing process of catfish fillets. Key findings: Ice crystals first appear in the four corners of the fillet because the temperature drop rates in these areas are higher than in the center. The freezing time predicted agreed well with the measured data.			
[83]	Crab meat and crab claws	COMSOL	2D	Triangular
	Study purpose: To simulate the freezing process of crab meat in pouches as well as the freezing process of crab claws taking into account the irregular shape. Key findings: The warmest spots inside the products did not remain in fixed positions and their positions changed with time. The numerical models could predict the freezing time and reproduce the time–temperature curves in the products accurately. The root-mean-square error (RMSE) and maximum absolute deviation (MAD) between the predicted and measured crab meat temperature were 1.5 °C and 3.65 °C, respectively. For the crab claws the RMSE was 0.99 °C and the MAD was 2.65 °C.			
[84]	Beef	Mathematical modeling	3D	NA
	Study purpose: To use a FEM to analyze the freezing process for frozen food processing, predict the freezing time at different freezing conditions and investigate the effect of freezing parameters on the freezing process. Key findings: It is proposed that the food shape and size, freezing air temperature and freezing air velocity are the most important factors affecting freezing rate. Furthermore, freezing parameters should be matched with the thermal conductivity of the given food.			
[85]	Bakery products	SOLIDWORKS	3D	Tetrahedral
	Study purpose: To develop and validate a code to simulate the freezing process of an irregularly shaped food using a combined enthalpy and Kirchhoff transformation method. Key findings: The numerical simulation was validated with analytical solutions and compared with experimental time–temperature curve using bakery products, and good agreement was reported. Additionally, the thicker zone of the products reached lower temperatures more slowly. The numerical model well predicted the time–temperature evolution of the freezing process. The maximum absolute error was lower than 1.3 °C.			
[86]	Mushrooms	MATLAB	3D	Tetrahedral/1882 nodes and 7693 elements
	Study purpose: To predict the freezing time of mushrooms considering the actual shape of the product. Key findings: The corners of the mushrooms reached lower temperatures faster than the thicker central areas. The numerical simulations agree with the experimental time–temperature curves well for the freezing of mushrooms. The maximum absolute error was lower than 3.1 °C.			
[87]	Breakfast box containing peppers, onions, orange juice, French toast, beefsteaks, and danishes	COMSOL	3D	Tetrahedral/23,019 domain elements, 7221 boundary elements and 1012 edge elements
	Study purpose: To investigate the effect of environmental conditions on the thermal behavior of a breakfast menu box during storage and transportation. Key findings: The corner areas of each food began to thaw first. Depending on the initial freezing point, the latent of phase change, the position, orientation, and other thermo-physical features. The order in which the food items thawed are: danishes, orange juice, peppers, onions, French toast, and beefsteak. The average deviations between the numerically simulated and experimentally obtained temperature data were 2 °C and 1 °C during freezing and thawing, respectively.			
[88]	Cylindrical ginger	ANSYS	1D	Brick/19856 elements
	Study purpose: To predict the freezing time of the product for two different freezing methods. Key findings: Simulated freezing temperature-time curves were in good agreement with the measured results (r = 0.97 for slow freezing and r = 0.92 for quick freezing). Slow and fast freezing at temperatures <−5 °C resulted in freezing time differences of 9 min and 2 s, respectively between the prediction method computed freezing rate and those from experiments.			
[89]	Brussels sprouts	COMSOL	3D	Triangular/4664 elements and 2437 nodes
	Study purpose: To predict the time–temperature histories and freezing times as a function of the surface heat transfer coefficient, refrigerant fluid temperature, and initial temperature of the product. Key findings: The proposed model was able to predict the time–temperature evolution during the freezing process successfully. The RMSE of the predicted temperatures histories was 0.965 °C.			
[90]	Mozzarella cheese	COMSOL	3D	Tetrahedral
	Study purpose: To model the freezing of cheese with FEM coupled with a photogrammetric approach that permits reconstructing the 3D domain of the non-regular spheroidal shaped cheese. Key findings: The model proposed that a surface-area-to-volume ratio ranging between 1.09 and 1.15 cm^−1^ was the most critical parameter that defines the freezing time of the product. Experimental temperature evolution observations were in good agreement with the numerical simulations. The RMSE and MAD were lower than 1.47 °C and 3.4 °C, respectively.			
[91]	Methylcellulose gels	COMSOL	Hybrid 2D–3D	Tetrahedral/17,672 elements
	Study purpose: To develop a numerical model capable of simulating the microwave-assisted freezing process. The phase change of the model was based on an enthalpy formulation and the growth of the spherical ice. Key findings: The dimensions of the sample, especially height and width, could affect the thermal homogeneity of the product during microwave-assisted freezing. The simulated temperature curve was very close to the average measured curve and the difference was about 1 °C.			
[92]	Dual tylose/water system	MATLAB	3D	Linear tetrahedral/1879 elements and 491 nodes
	Study purpose: To develop a multi-optional FE code to solve the enthalpy and Kirchhoff transform heat conduction equation. Key findings: The proposed solution greatly reduced the execution speed compared to traditional FE formulations based on the original non-linear or enthalpy-transformed Fourier equation. The RMSE between the predicted and measured temperatures was 1.2 °C.			
[93]	Methylcellulose gels	COMSOL	2D	NA
	Study purpose: To explore the thermal interactions between a product being frozen and microwaves in a microwave-assisted freezing system. Key findings: The microwave behavior in the sample was strongly influenced by the freezing front location and the air–product interfaces.			
[94]	Sucrose solutions	COMSOL	2D	NA
	Study purpose: To predict the evolution of velocity, temperature, pressure and ice fraction of product at each point of the scraped surface heat exchanger. Key findings: Recirculation areas were observed between the scraping blades.			
[95]	Sorbet	COMSOL	3D	Prismatic (around the surface) and tetrahedral (interior parts)/1.5 × 10^6^ elements
	Study purpose: To solve the coupled problem of fluid flow and heat transfer in a scraped surface heat exchanger during the production of sorbet. Sensitivity analysis was performed to assess the influence of key model parameters (heat transfer coefficient at the exchanger inner wall and thermal conductivity of the solid elements (dasher and blades)) on the model predictions. Key findings: The highest velocities occurred near the exchanger wall. Experimental temperature evolution observations along the heat exchanger were in good agreement with the numerical simulations. The influence of the heat transfer coefficient at the exchanger wall on the predicted temperature was relatively high and the influence of conduction in solids was relatively weak.			

**Table 2:** Examples of finite element method application in food thawing.

Reference	Material	FEM Analysis Tool	Dimension	Resolution (type/size)
[10]	Beef meat	COMSOL	3D	NA
	Study purpose: To simulate the tempering process of frozen beef with selected sizes and shapes. Key findings: As the thickness of the sample increased, and the base area decreased, the heating rate increased and the heating uniformity decreased. The cuboid shape had the best heating uniformity, followed by trapezoidal prism and step shape. The computer simulation results of temperature distribution agreed well with the experimental results.			
[47]	Packed peas, spinach cubes and grilled aubergines	COMSOL	3D	Tetrahedral/6947 to 507,227 elements
	Study purpose: To study the influence of environmental temperature on heat transfer inside frozen foods using validated FEM models. A sensitivity analysis was performed to understand the influence of five mesh size intervals ranging from 0.00005 to 0.025 m on simulation time and product core temperature. Key findings: Thawing started from the bottom of the product in contact with the metallic rack. Simulation results indicated that the mesh grid size had a significant effect on the simulation time and computed food core temperature. Considering the core temperature, a good agreement was observed between simulated and experimental results. A maximum error (ME) of 0.8 °C, −1.7 °C, and −0.5 °C and root-mean-square error (RMSE) of 0.3 °C, 0.6 °C, and 0.2 °C were achieved for grilled aubergines, spinach cubes and peas, respectively. The accuracy of temperature prediction in the bottom zone of the products was slightly lower. ME values of 1.8 °C, −1.7 °C, and 1.5 °C and RMSE of 0.6 °C, 0.8 °C, and 0.6 °C were achieved for grilled aubergines, spinach cubes, and peas, respectively.			
[108]	Minced fish block	COMSOL	3D	NA
	Study purpose: To investigate the characteristics and optimal conditions RF thawing by elucidating the temperature distributions in blocks of frozen minced fish. Key findings: The best electrode distance for the most common sample size used in industry (25 × 15 × 5 cm^3^) was found to be 16 cm because it provided a more uniform temperature distribution and better gel strength. Increasing the block’s bottom area could reduce the edge effect of the electromagnetic field.			
[114]	Congee with minced pork	COMSOL	3D	Free tetrahedral (air domain) and quadrilateral (frozen sample)
	Study purpose: To study the effects of power input and food aspect ratio on microwave thawing process of frozen food using FEM. Key findings: Microwave power density and sample size affected heating uniformity and thawing time. The RMSE values of transient temperature ranged from 6.14 and 12.88 °C depending on the measurement locations.			
[115]	Tylose cube	COMSOL	3D	Tetrahedral
	Study purpose: To evaluate the effect of continuous change of dielectric properties of frozen material on microwave power absorption during heating. Key findings: Energy absorption decreased as the product thawed. The lowest and highest temperatures were observed near the center of the product and the edges, respectively. The salt concentration had a significant effect on changing the electrical properties of the sample but did not significantly alter the heating rate.			
[117]	Shrimp	COMSOL	3D	NA
	Study purpose: To simulate temperature distribution and thawing time of shrimp during ultrasound-assisted thawing and the influence of thawing process on protein denaturation. Key findings: Ultrasound-assisted thawing compared with water immersion enhanced thawing rate and reduced thawing time by 35.9%. Proteins with molecular mass from 70 to 100 kDa were degraded and cross-linked after thawing. The RMSE of internal temperature of sample between the predicted and measured values was 0.90 °C for water immersion thawing, and 0.94 °C for ultrasonic-assisted thawing.			
[118]	Lean beef	COMSOL	3D	Lagrange-quadratic/286,000 elements
	Study purpose: To simulate the electrical field distribution inside a RF system and the temperature distribution in the frozen product during thawing. Key findings: The simulation results were consistent with experimental data. The RMSE of the simulated temperature ranged from 0.9 to 1.3 °C, depending on the distance between the sample surface and the upper electrode.			
[122]	Dual water/soy oil system	Photo-Wave-jꞷ	3D	NA
	Study purpose: To investigate the power absorption of two-component materials during microwave thawing. Key findings: The absorbed power during the microwave thawing process was very different with irregular increases and decreases in the absorbed power amounts. In low-volume samples (≤200 mL) the total absorbed power increased with increasing water, especially in the water regions. In large-volume samples (≥500 mL), the oil regions absorbed more power compared to the same ratio composition in low-volume samples.			
[131]	Tuna muscle	Photo-Wave-jꞷ	3D	Brick/5 mm mesh spacing
	Study purpose: To model the RF defrosting of tuna by incorporating heat transfer analysis and electromagnetic field analysis. Key findings: The model was able to predict transient temperature profiles. A more uniform heat distribution was obtained when the electrode and the upper surface of the sample were the same size. The difference between the simulated and measured temperature profiles was small.The absolute values of relative errors at the central and corner positions of tuna muscle were 4.6% and 2.1%, respectively.			
[132]	Mashed potato	COMSOL	3D	Tetrahedral/546,853 (entire domains) and 134,285 (mashed potato) elements
	Study purpose: To develop a procedure incorporating electromagnetic frequency spectrum into coupled electromagnetic and heat transfer model for accurate temperature predictions during microwave thawing. Key findings: Incorporating a 0.05 GHz standard deviation into the microwave frequency improved the prediction accuracy of the transient temperature profile and temperature field pattern compared to the assumption of a constant 2.45 GHz frequency. In the transient temperature profile measurement, the average RMSE value was 13.1 °C and 7.5 °C for simulations using monochromatic frequency of 2.45 GHz and frequency spectrum, respectively.			
[133]	Mashed potato	COMSOL	3D	Tetrahedral and prismatic/546,960 (entire domains) and 190,985 (food domain) elements
	Study purpose: To develop a model for microwaving mashed potatoes incorporating electromagnetic, heat and mass transfer Darcy’s velocity as well as phase change of melting and water evaporation. Key findings: After 2 min of microwave heating, hot spots were observed on the edges and a hot ring around the center of the top layer of the sample. As process time progressed, hotspots developed from the edges to the center.The average RMSE values of total moisture loss and transient temperature were less than 2.4 g and 13.2 °C, respectively.			
[134]	Large tuna fishes	COMSOL	3D	Tetrahedral/1,312,939 elements
	Study purpose: To develop a numerical model to study water immersion thawing process of the product.Key findings: The FEM results showed that it was not necessary to consider the internal details of the fish components to simulate the temperature distribution and that the reconstruction of the external contours was sufficient. Ambient temperature strongly affected the thawing time. Good agreement was found between the measured and simulated temperatures.			
[135]	Chinese fast foods	COMSOL	3D	Free tetrahedral
	Study purpose: To simulate the microwave heating process and evaluate the rotation speed of the turntable on the microwave heating distribution. Key findings: The best rotation speed for the product inside the microwave oven was 7.5 rpm. The experimental spatial temperature profile was in good agreement with the modeled spatial temperature profile. The RMSE values were 1.30–2.86 °C in chicken nuggets and 1.56 °C and 7.68 °C in Chinese steamed bread depending on the measurement locations.			
[136]	Pork products	MATLAB	2D	NA
	Study purpose: To simulate the temperature distribution in the product during tempering process and to explore the effects of external (convective heat transfer coefficient and ambient temperature) and internal (size and composition) parameters on tempering time. Key findings: The composition and thickness of the product affected the tempering time. The maximum time required to complete thawing occurred when the ambient temperature was approximately 1 °C higher than the freezing point of the product.			

## Data Availability

Not applicable.
